# Challenging the Interpretation of White Blood Cell Counts in Patients with Sepsis Following Packed Cell Transfusion †

**DOI:** 10.3390/jcm12123912

**Published:** 2023-06-08

**Authors:** Moti Klein, Lior Hassan, Rivka Katz, Ran Abuhasira, Matthew Boyko, Ohad Gabay, Dmitry Frank, Yair Binyamin, Victor Novack, Amit Frenkel

**Affiliations:** 1 General Intensive Care Unit, Soroka University Medical Center, and The Faculty of Health Sciences, Ben-Gurion University of the Negev, Beer-Sheva 7747629, Israel; 2 The Joyce and Irving Goldman Medical School, Faculty of Health Sciences, Ben-Gurion University of the Negev, Beer-Sheva 7747629, Israel; 3 Clinical Research Center, Soroka University Medical Center, and The Faculty of Health Sciences, Ben-Gurion University of the Negev, Beer-Sheva 7747629, Israel; 4 Department of Anesthesiology, Soroka University Medical Center, The Faculty of Health Sciences, Ben-Gurion University of the Negev, Beer-Sheva 7747629, Israel; 5 Anesthesia, Critical Care and Pain Medicine, Beth Israel Deaconess Medical Center, Harvard Medical School, Boston, MA 02115, USA

**Keywords:** intensive care unit, white blood cells count, infection, sepsis, leukocytosis

## Abstract

Critically ill patients with sepsis often require packed cell transfusions (PCT). However, PCT may affect white blood cell (WBC) counts. We conducted a population-based retrospective cohort study to trace changes in WBC count following PCT in critically ill patients with sepsis. We included 962 patients who received one unit of PCT while hospitalized in a general intensive care unit, and 994 matched patients who did not receive PCT. We calculated the mean values of WBC count for the 24 h before and 24 h after PCT. Multivariable analyses using a mixed linear regression model were performed. The mean WBC count decreased in both groups, but more in the non-PCT group (from 13.9 × 10^9^/L to 12.2 × 10^9^/L versus 13.9 × 10^9^/L to 12.8 × 10^9^/L). A linear regression model showed a mean decrease of 0.45 × 10^9^/L in WBC count over the 24 h following the start of PCT. Every 1.0 × 10^9^/L increase in the WBC count prior to PCT administration showed a corresponding decrease of 0.19 × 10^9^/L in the final WBC count. In conclusion, among critically ill patients with sepsis, PCT causes only mild and clinically non-prominent changes in WBC count.

## 1. Introduction

Various factors can increase or decrease white blood cell (WBC) count, resulting in leukocytosis or leukopenia, respectively. Leukocytosis can result from multiple causes, including infections, inflammation, bone marrow disorders, and certain medications. Conversely, autoimmune diseases and certain bacterial and viral infections often lead to leukopenia [1,2,3]. Monitoring WBC count is a simple, inexpensive, and readily available tool for patient assessment. Thus, WBC monitoring is integral to daily routine blood work in clinical practice, and specifically for critically ill patients hospitalized in intensive care units (ICU). The frequency of WBC measurement varies between institutions, but twice daily is generally accepted [4]. Monitoring WBC count serves as a means of evaluating new infections in critically ill patients, such as line sepsis or ventilator-associated pneumonia [5], and of evaluating the status of the infection that is the primary reason for the patient’s hospitalization in the ICU. Variations in WBC count can have not only clinical but also economic implications. A misinterpretation of WBC count may result in unnecessary prolonged hospital stays and additional treatments, leading to increased healthcare costs. For instance, in a patient with pneumonia, leukocytosis may persist despite several days of treatment, leading physicians to extend antibiotic treatment incorrectly [6], when in fact another misleading factor has emerged that prolongs the leukocytosis [7]. Another example highlighting the economic burden involves the unnecessary initiation of antibiotic treatment in a patient with leukocytosis due to an obscured distracting factor. As a result, a decision is made to replace a central catheter and begin antibiotic treatment, believing the existing catheter to be contaminated.

Low levels of hemoglobin are common in critically ill patients, and often require packed cell transfusions (PCT) of red blood cells [8]. PCT contains various immunomodulatory mediators that can interact with immune cells, leading to both proinflammatory and immunosuppressive effects [9], and resulting in substantial changes in WBC count [10]. Limited data exist regarding the effect of PCT on WBC count among critically ill patients with sepsis. In a prospective, non-interventional study of 96 patients, Izbicki et al. [11] measured WBC count before and 2, 4, 6, 12, and 24 h following PCT. Post-transfusion leukocytosis was found to occur in 76% of non-septic compared to only 15% of septic patients (*p* < 0.001). In the septic group, significant post-transfusion leukocytosis was not observed. However, the study included only 20 patients with sepsis, used time intervals that are not acceptable in clinical practice for measuring WBC count, and lacked statistical correction for possible confounders of WBC count.

Following from the above, PCT potentially alters WBC count and confuses the interpretation of WBC count changes, particularly of new bacteremia events in critically ill patients. Thus, the primary objective of this study was to track WBC count changes during the 24 h following PCT in critically ill patients with sepsis.

## 2. Materials and Methods

### 2.1. Study Population

This population-based retrospective case–control study included patients who were hospitalized during the years 2000–2019 in the ICU at Soroka University Medical Center. This tertiary care medical center is the only regional hospital in southern Israel, namely, the Beer-Sheva vicinity, with an estimated population of 1,000,000. Study eligibility criteria were age 18 years or older and the presence of sepsis or septic shock.

We collected data of patients who received no more than one unit of PCT within 24 h. The following data were accessed: demographics (age, sex), hospitalization details (length of stay), underlying medical conditions according to the chronic disease registries of Clalit Health Services, and laboratory blood results from the index hospitalization. The blood samples utilized in the study were not specifically obtained for research purposes; rather, we employed blood samples collected from patients as part of routine testing procedures. The test tubes employed in the study were equipped with Ethylenediaminetetraacetic acid (EDTA) as a chelating agent. These samples were transported to the hematology laboratory at Soroka Medical Center and promptly inserted into an ADVIA2100i device. In light of the documented impact of systemic steroids on raising white blood cell (WBC) count [12], we have specifically excluded patients who were administered steroids during their hospital stay. The outcomes of the analysis were obtained within a timeframe of 5–10 min and subsequently electronically transmitted to the hospital’s information systems.

**Figure 1:**
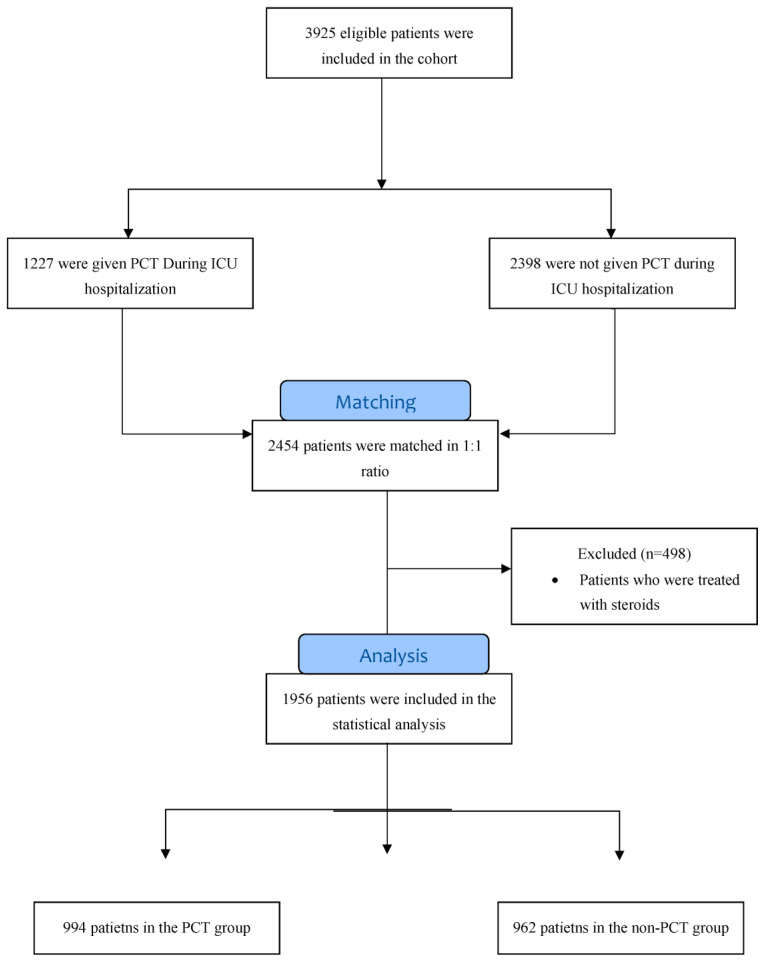
Consort diagram of study population.

A control group was formed by pairing eligible patients with other patients who had been admitted to the general ICU with sepsis or septic shock and who did not receive PCT during their ICU stay. These patients were matched by age (±3 years), sex, and socio-economic status as indicated by the “Social State Score”. This measure is used to classify individuals and families into socioeconomic categories based on their sources of income, education level, occupation, and lifestyle. Figure 1 (a consort diagram) presents the patient flow chart, according to the study inclusion criteria.

### 2.2. Primary Exposure and Outcome Assessment

The hospitalization day of the PCT was used to calculate the delta for the non-PCT group. To evaluate the relation between PCT and the changes in WBC, multivariable analyses using mixed linear regression were performed. We calculated the mean values of WBC, 24 h before and after PCT administration for the exposed group. To calculate the delta WBC for the non-exposed group, we employed a method wherein each subsequent measurement served as a reference point for establishing the baseline measurement. Specifically, the baseline measurement for each individual was determined by considering the WBC count at the previous measurement time point, enabling us to calculate the change in WBC count over the duration of the study. The statistical analyses were performed using R-studio, version 1.1.423, Rstudio, Inc., Boston, MA, USA. A *p*-value of less than 0.05 was considered statistically significant.

### 2.3. Statistical Analysis

Descriptive statistics are provided using summary tables. The statistics for continuous normal distribution variables included means and standard deviations. Continuous variables that were not normally distributed included medians and interquartile ranges. Categorical variables are described with numbers and percentages. Comparisons between groups are presented by *p*-values. Percentages were rounded to one decimal place.

Parametric model assumptions were assessed using a normal plot or Shapiro–Wilk statistic for verification of normality, and Levine’s test for verification of homogeneity of variances. For continuous variables, the paired samples *t*-test and Wilcoxon signed-rank test were used as non-parametric procedures if parametric assumptions could not be satisfied, even after attempts at data transformation, or for ordinal variables. Categorical variables were tested using the McNemar test. All *p*-values reported were rounded to three decimal places. Variables were selected in multivariable modeling, based on clinical and statistical significance. First, baseline clinical (diagnoses coded in ICD-9) and demographic characteristics (age, sex) were selected, then clinical factors (co-morbidities). We used linear mixed model to evaluate the association between PCT and WBC levels.

## 3. Results

### 3.1. Study Population

The study population comprised 962 patients who received one unit of PCT, and 994 matched patients. Table 1 summarizes their characteristics. The majority (57%) of patients were males. Hypertension was the most common comorbidity, present in 52% of both the PCT and non-PCT groups.

### 3.2. Characteristics of the Hospitalization Course (Table 2)

Compared to the non-PCT group, for the PCT group the ICU stay was prominently longer, more patients received vasopressors, and the 30-day post-admission mortality rates were higher.

**Figure 2:**
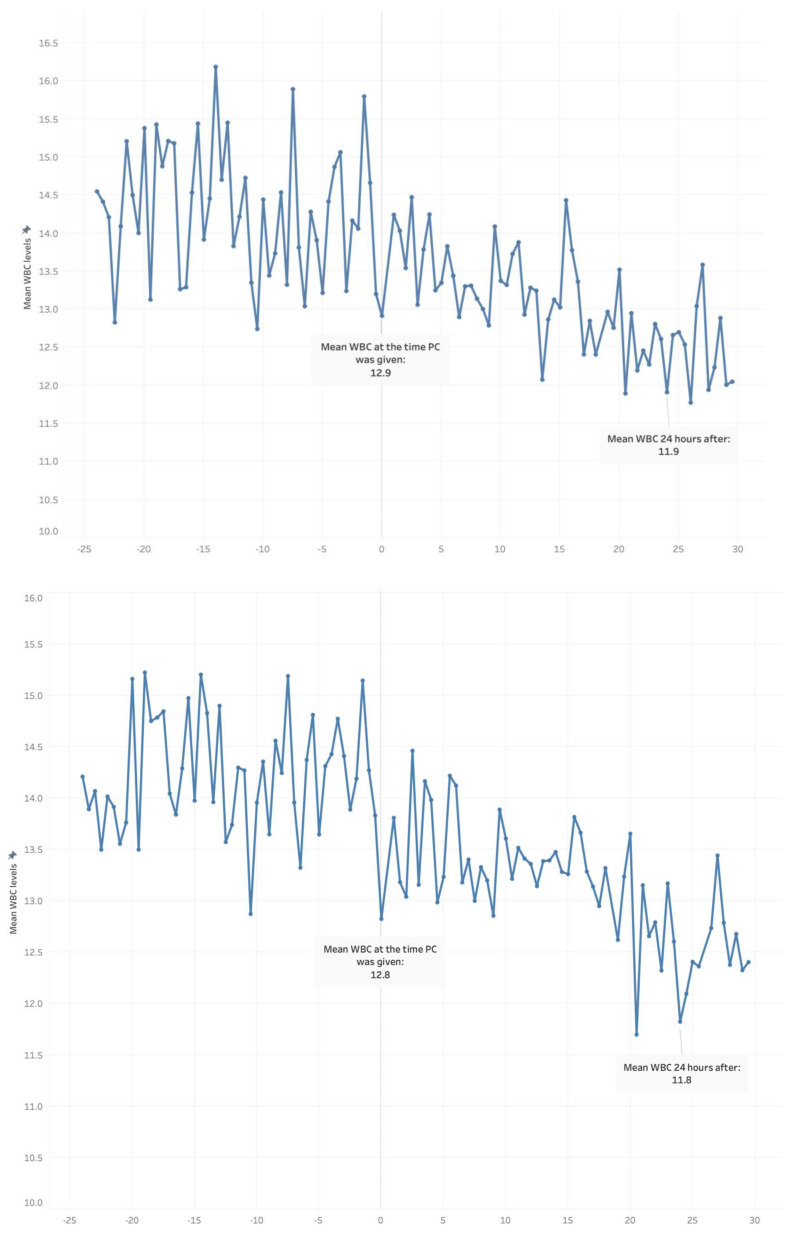
The change in mean white blood cell (WBC) count before and after packed cell transfusion (PCT).

Figure 2 presents the changes in WBC count around the time of administration of the single PCT. The mean WBC count within the 24 h before PCT was 14.5 × 10^9^/L, and decreased to a mean 12.9 × 10^9^/L by the time of transfusion. The mean WBC count continued to drop, to a low of 11.9 × 10^9^/L at 24 h after the transfusion. In all the patients who received PCT, no abnormal reaction was observed, including a febrile non-hemolytic reaction.

**Table 2:** Clinical characteristics of patients with sepsis hospitalized in a general intensive care unit, according to receipt of packed cell transfusions.

	Non PCT Group N = 994	PCT Group N = 962	*p*-Value
ICU hospitalization days—Median (IQR)	3 (2, 5)	8 (3, 20)	<0.001
30-day post-admission mortality rate	181 (18%)	246 (26%)	<0.001
Vasopressor given during ICU stay—n (%)	256 (26%)	527 (55%)	<0.001
Mechanical ventilation—n (%)	994 (100%)	962 (100%)	1
ICU hospitalization SOFA score—Median (IQR)	5.5 (3.5, 7.6)	7.2 (5.7, 9.3)	<0.001
Initial Mean (SD) WBC count	13.9 × 10^9^/L (7.0)	13.9 × 10^9^/L (7.2)	0.813
Final Mean (SD) WBC count	12.2 × 10^9^/L (5.9)	12.8 × 10^9^/L (7.2)	<0.001

PCT, packed cell transfusion; ICU, intensive care unit; SD, standard deviation.

### 3.3. Linear Regression Model

**Figure 3:**
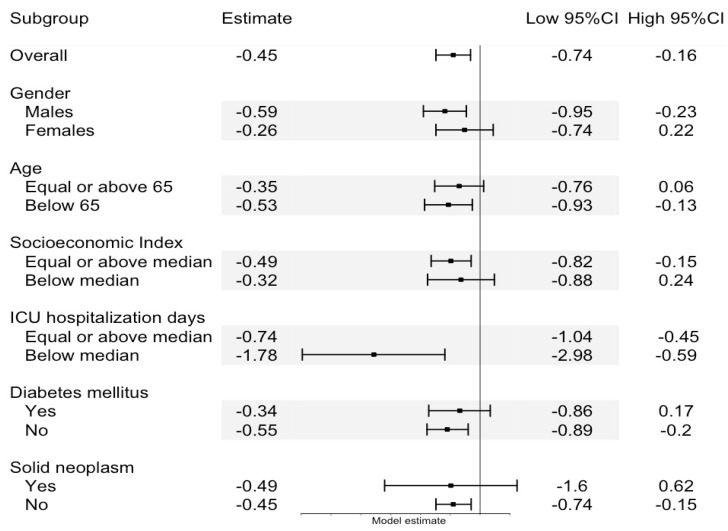
Linear mixed model subgroup analysis.

Table 3 presents the findings of the multivariable analysis that explored the relations between PCT administration, initial WBC count, age, and subsequent changes in WBC count. Among the patients who received a single unit of PCT, the mean WBC count decreased by 0.45 × 10^9^/L over the 24 h following the start of the transfusion. Additionally, for every 1.0 × 10^9^/L increase in the WBC count prior to PCT administration, the analysis revealed a corresponding decrease of 0.19 × 10^9^/L in the final WBC count. Furthermore, among the patients who received PCT, for every additional year of age, the mean WBC increased by 0.02 × 10^9^/L. Figure 3 presents the linear mixed model subgroup analysis.

## 4. Discussion

The main finding of our study is that the administration of a single unit of PCT in critically ill patients caused a mean mild decrease in the WBC count of 0.45 × 10^9^/L. We also report an inverse relation between the WBC count values before and after the infusion. Accordingly, the higher the WBC count before the infusion, the greater the magnitude of the decrease in the WBC count following the infusion.

WBC count is a widely available, inexpensive, and common test used to predict bloodstream infections, as well as to follow known infections [13]. Changes in WBC count following PCT have already been demonstrated, also in the context of predicting prognosis in the setting of sepsis. A study published in 2022 by Emily Rimmer et al. [14] found that analyzing WBC trajectories in patients with sepsis can reveal distinct and clinically relevant sub-populations. They reported an association between a rising WBC trajectory and higher mortality. This highlights the importance of investigating and understanding factors that may affect WBC count, such as PCT. The finding can help clinicians distinguish variations in WBC count that appear as confounders rather than as major events requiring therapeutic attention.

The current study demonstrated clinically negligible changes in WBC count following PCT. The small decrease in the WBC count was apparent both in the univariate analysis and in the multivariate analysis. The initial WBC count and final WBC count, before and after PCT, respectively, were both elevated, consistent with the expectation of leukocytosis in patients with sepsis. The likelihood is thus low that such changes will mislead the treating staff, and cause them to respond as if a clinically important event appeared. Notably, a parallel conclusion was previously reported by Klein et al. [15] in the same population. In that study of critically ill patients with sepsis, PCT itself caused mild temperature change, without clinical significance. A consequent insight from the results of the current study is that the appearance of significant changes in WBC count during the 24 h following PCT may indicate an unusual clinical event that requires immediate attention. A classic example of this, in the setting of critically ill patients, would be a new peak in leukocytosis. In a ventilated critically ill patient, for example, this could raise suspicion of ventilator-associated pneumonia. Consequently, a cascade of diagnostic procedures may ensue, including chest X-ray examinations, and potentially invasive interventions such as bronchoalveolar lavage. These measures are commonly employed in the diagnosis of ventilator-associated pneumonia [16,17], and their implementation depends on the particular protocols followed within the intensive care unit (ICU).

Our study has a number of limitations, including its single-center retrospective design. Notably, the study focused on the WBC count without inspecting the leukocyte subtypes (lymphocytes, neutrophils, etc.). Thus, further examination of the leukocyte subpopulation could possibly have revealed a clinically significant change in a particular leukocyte, despite the lack of a significant change in the general WBC count.

Only a few studies have focused on the effect of changes in WBC count following PCT in critically ill patients with sepsis. To the best of our knowledge, this is the first investigation that performed statistical correction aimed to eliminate the effects of other possible confounders on the WBC count. Our study primarily focused on the alterations in WBC count following the administration of PCT. However, future research directions may include investigating the impact of PCT on other laboratory parameters commonly used by physicians to monitor infectious status, such as the count of neutrophils that rise significantly during bacterial infections and the levels of C-reactive protein. These research directions would help us to extend our understanding of “laboratory changes following PCT” and improve patient care.

## 5. Conclusions

We suggest that PCT administration causes a clinically negligible change in WBC count in critically ill patients with sepsis. Therefore, clinicians should interpret substantial changes in WBC count (leukocytosis or leukopenia) during the 24 h following PCT, as potential indicators of unusual clinical events requiring attention.

## Figures and Tables

**Table 1:** Demographic and clinical characteristics of the study population.

	Non-PCT Group N = 994	PCT Group N = 962	*p*-Value
Sex—n (%)			0.986
Male	571 (57%)	553 (57%)	
Age—Mean (SD)	59 (21)	59 (21)	0.938
LMS Social State Score—Median (IQR)	3.00 (2.00, 5.00)	3.00 (2.00, 5.00)	0.208
Hypertension	517 (52%)	501 (52%)	0.976
Myocardial infraction—n (%)	167 (17%)	175 (18%)	0.418
Congestive heart failure—n (%)	138 (14%)	148 (15%)	0.347
COPD—n (%)	223 (22%)	209 (22%)	0.705
Diabetes mellitus—n (%)	325 (33%)	305 (32%)	0.639
Solid neoplasm—n (%)	61 (6.1%)	61 (6.3%)	0.852

PCT, packed cell transfusion; SD, standard deviation; IQR, interquartile range; COPD, chronic obstructive pulmonary disease.

**Table 3:** Multivariable Analysis: Findings from a Mixed Linear Model on the Alteration of White Blood Cell Count following Packed Cell Transfusion.

Predictors	Estimates	CI	*p*
PCT given during ICU hospitalization	−0.45	−0.74–−0.16	0.002
WBC count before PCT	−0.19	−0.21–−0.17	<0.001
Age	0.02	0.01–0.02	<0.001

PCT, packed cell transfusion; ICU, intensive care unit; WBC, white blood cell.

## Data Availability

The data used in the analysis of this study are not publicly available due to national regulations, but are available from the corresponding author upon request and following the Ethics Committee approval.
